# Treatment of Recurrent Posttransplant Lymphoproliferative Disorder of the Central Nervous System with High-Dose Methotrexate

**DOI:** 10.1155/2013/765230

**Published:** 2013-08-01

**Authors:** Clare J. Twist, Ricardo O. Castillo

**Affiliations:** ^1^Division of Hematology-Oncology, Department of Pediatrics, Lucile Salter Packard Children's Hospital, Suite 300, 1000 Welch Road, Palo Alto, CA 94304, USA; ^2^Division of Gastroenterology, Department of Pediatrics, Lucile Salter Packard Children's Hospital, Palo Alto, CA 94304, USA

## Abstract

Posttransplant lymphoproliferative disorder (PTLD) is a frequent complication of intestinal transplantation and is associated with a poor prognosis. There is currently no consensus on optimal therapy. Recurrent PTLD involving the central nervous system (CNS) represents a particularly difficult therapeutic challenge. We report the successful treatment of CNS PTLD in a pediatric patient after liver/small bowel transplantation. Initial immunosuppression (IS) was with thymoglobulin, solucortef, tacrolimus, and mycophenolate mofetil. EBV viremia developed 8 weeks posttransplantation, and despite treatment with cytogam and valganciclovir the patient developed a polymorphic, CD20+, EBV+ PTLD with peripheral lymphadenopathy. Following treatment with rituximab, the lymphadenopathy resolved, but a new monomorphic CD20−, EBV+, lambda-restricted, plasmacytoid PTLD mesenteric mass emerged. Complete response of this PTLD was achieved with 6 cycles of cyclophosphamide, doxorubicin, vincristine, and prednisone (CHOP) chemotherapy; however, 4 months off therapy he developed CNS PTLD (monomorphic CD20−, EBV+, lambda-restricted, plasmacytoid PTLD) of the brain and spine. IS was discontinued and HD-MTX (2.5–5 gm/m^2^/dose) followed by intrathecal HD-MTX (2 mg/dose ×2-3 days Q 7–10 days per cycle) was administered Q 4–7 weeks. After 3 cycles of HD-MTX, the CSF was negative for malignant cells, MRI of head/spine showed near-complete response, and PET/CT was negative. The patient remains in complete remission now for 3.5 years after completion of systemic and intrathecal chemotherapy. *Conclusion*. HD-MTX is an effective therapy for CNS PTLD and recurrent PTLD that have failed rituximab and CHOP chemotherapy.

## 1. Introduction

PTLD of the CNS is a rare complication of solid organ transplantation (SOT) and there is currently no consensus on optimal therapy. Recurrent PTLD following rituximab and front-line chemotherapy represents a particularly difficult therapeutic challenge. We report the successful use of HD-MTX and intrathecal MTX to treat recurrent PTLD of the CNS in pediatric patient status after combined liver/small bowel transplantation. 

## 2. Case Report

**Figure 1:**
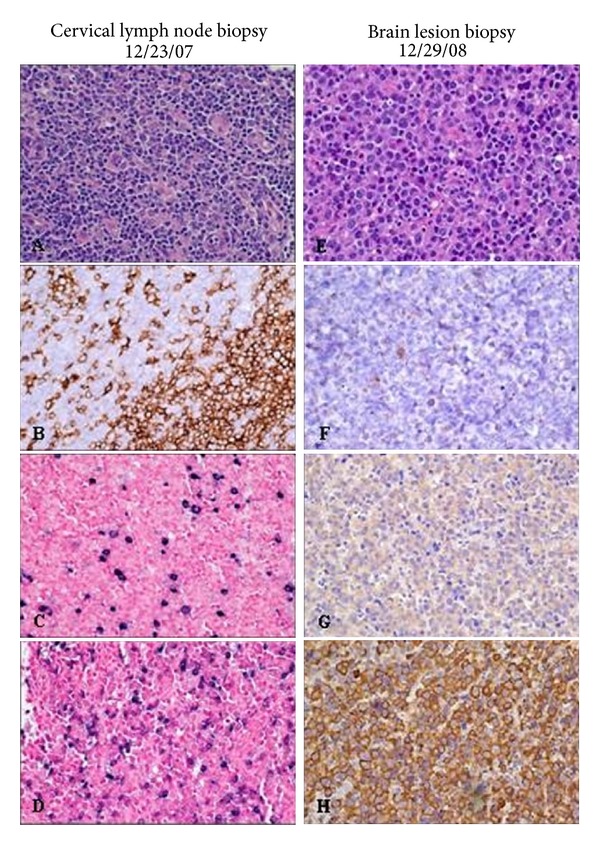
(A) Polymorphic PTLD. The nodal parenchyma is mostly effaced by a mixed population of small and large lymphocytes, immunoblasts, and plasma cells. (B) CD20 immunochemistry shows reactivity in residual follicles, as well as in scattered larger B cells outside of follicles. (C) *In situ* hybridization for kappa light chains shows scattered expression in plasma cells. (D)* In situ *hybridization for lambda light chains shows mild predominance of lambda expressing plasma cells over kappa. (E) Monomorphic PTLD: many cells resemble immunoblasts and plasmablasts with large nuclei, prominent nucleoli, and eccentric pink cytoplasm. (F) CD20 immunochemistry shows minimal expression, as CD20 is typically lost as B cells differentiate towards plasma cells. (G) Immunohistochemical stain for kappa light chains shows weak, nonspecific reactivity. (H) Immunohistochemical stain for lambda light chains shows strong cytoplasmic reactivity.

Our patient underwent combined liver/small bowel transplantation at the age of 15 months for short bowel syndrome secondary to ileal atresia and TPN-associated liver disease. Donor and recipient were both CMV and EBV seronegative. Immunosuppression (IS) was with thymoglobulin, solucortef, tacrolimus, and mycophenolate mofetil (MMF). MMF was discontinued after two weeks due to increased ostomy output. EBV viremia developed at 8 weeks posttransplantation, and despite treatment with cytogam and valganciclovir, the patient developed widespread lymphadenopathy. Cervical lymph node biopsy revealed a polymorphic, CD20+, EBV+ PTLD (Figures 1(A) and 1(B)). Kappa and lambda immunochemistry and *in situ* hybridization studies showed a mild predominance of lambda-expressing over kappa-expressing plasma cells, but no definite light chain restriction within the B-cell or plasma cell populations (Figures 1(C) and 1(D)). Polymerase chain reaction for immunoglobulin heavy chain (IgH) gene rearrangements, however, demonstrated a small peak, consistent with the presence of a clonal B-cell population. PET/CT imaging confirmed FDG-avid lymphadenopathy in the cervical, axillary, mediastinal, and inguinal regions. 

**Figure 2:**
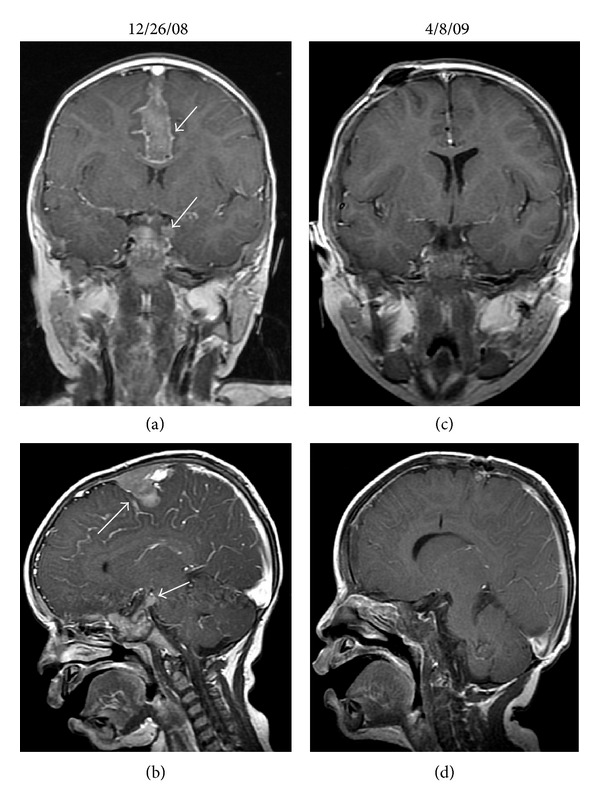
(a) 12/26/08 MRI coronal T1 postgadolinium image demonstrates a dural-based mass along the falx with heterogeneous enhancement (arrow). (b) 12/26/08 MRI sagittal T1 post-gadolinium image demonstrates the mass along the falx and an enhancing focus in the suprasellar cistern (arrows). (c) 4/8/09 MRI coronal T1 post-gadolinium image shows resolution of the previously identified enhancing mass within the falx. An Ommaya reservoir has been placed in the interim. (d) 4/8/09 MRI sagittal T1 post-gadolinium image shows resolution of previously seen enhancing anterior falx and resolution of the suprasellar mass.

**Figure 3:**
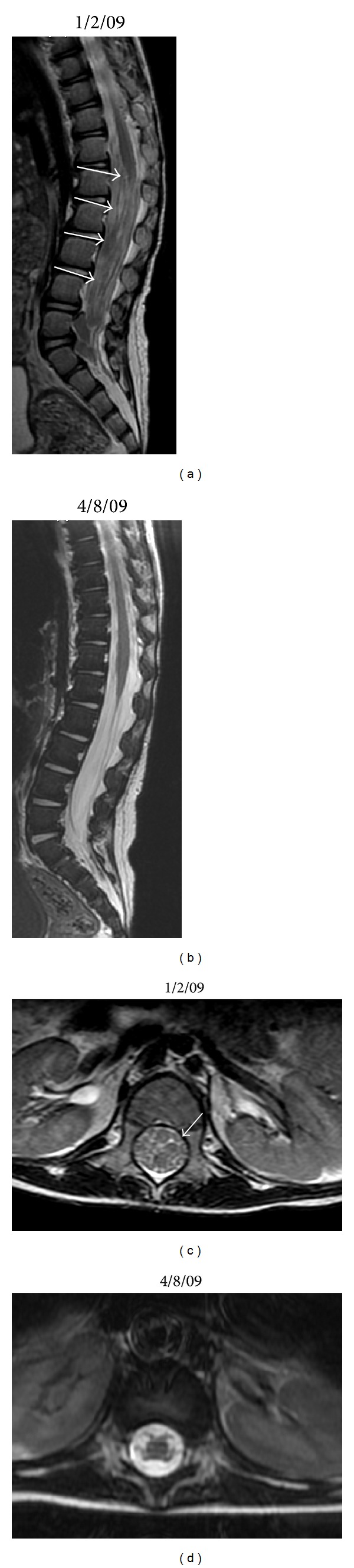
(a) 1/2/09 MRI spine T2 weighted image shows extensive abnormal and nodular enhancement filling the thecal sac from T11-12 extending down through the lumbosacral spine (arrows). (b) 4/8/09 MRI spine T2 weighted image shows near resolution of previously seen abnormal enhancement. (c) 1/2/09 MRI spine axial image demonstrates enhancing masses and thickened nerve roots filling the thecal sac at L1. (d) 4/8/09 MRI spine axial image with normal appearance of thecal sac and nerve roots at L1.

The patient was treated with reduction of IS and rituximab (375 mg/m^2^/dose IV weekly ×4 doses). PET/CT imaging at the completion of rituximab confirmed the resolution of the previously noted sites of disease; however, a new FDG-avid mesenteric mass was identified. Fine needle aspirate of the mesenteric mass revealed a monomorphic CD20−, EBV+, lambda light chain-restricted PTLD with plasmacytoid differentiation. Further IS reduction was complicated by acute graft rejection, which was treated with infliximab. Chemotherapy was initiated with cyclophosphamide, doxorubicin, vincristine, and prednisone (CHOP). Six cycles of CHOP were administered and the patient's course was complicated by episodes of febrile neutropenia, bacteremia, disseminated *Mycobacterium chelonae* infection, herpes simplex virus, (HSV1) stomatitis, and chemotherapy-induced colitis. At the completion of CHOP chemotherapy, a complete response was confirmed by PET/CT. However, 4 months later the patient presented with headaches and MRI of the head and spine revealed multiple dural-based lesions overlying the posterior frontal/parietal lobe and other areas of extra-axial enhancement and nodularity, as well as lumbar thecal sac and epidural spinal masses (Figures 2 and 3). Biopsy of a parietal lesion confirmed a monomorphic CD20−, EBV+, lambda-restricted, plasmacytoid PTLD. 

With the diagnosis of CNS PTLD, all IS was discontinued and treatment with HD-MTX was initiated. The first course of therapy consisted of HD-MTX (5 gm/m^2^/dose IV over 24 hours on day 1) and cytarabine (500 mg/m^2^/dose IV on day 2 followed by 80 mg/m^2^/hour continuous IV infusion ×48 hours). Metronomic intrathecal (IT) MTX (2 mg/dose administered IT Q day ×2-3 sequential days every 10 days) was administered via an Ommaya reservoir [1]. This treatment course was complicated by generalized seizures on the day following HD-MTX administration as well as by *E. coli* and vancomycin-resistant *Enterococcus faecium* (VRE) sepsis, HSV1 stomatitis, respiratory syncytial virus (RSV) bronchiolitis, and chemotherapy-induced colitis. Due to the significant toxicity with the combination of HD-MTX and cytarabine, 4 subsequent courses were dose-modified to consist only of HD-MTX (2.5 gm/m^2^/dose on day 1) and IT MTX (2 mg/dose IT ×2-3 days Q 10 days beginning on day 7–10 of each HD-MTX cycle). Cycles of HD-MTX were repeated approximately every 28 days. Therapy was still complicated by episodes of febrile neutropenia, bacteremia, and diarrhea, but these were of markedly reduced severity in comparison to these seen with the higher dose of MTX in combination with cytarabine. No renal or hepatic toxicity was observed. Following 2 cycles of this chemotherapy, the patient's CSF no longer revealed malignant cells and after 3 cycles MRI of brain and spine confirmed complete radiographic response (Figures 2 and 3) and PET/CT scan was negative. 

A complete response of CNS PTLD was achieved with HD-MTX and IT MTX. A total of 5 cycles of HD-MTX were delivered over 5 months. Following completion of systemic chemotherapy, additional 5 months of only intrathecal chemotherapy was given, initially with metronomic IT MTX (see above) and subsequently with IT cytarabine (15 mg/dose ×3 days, administered Q 21 days). He did not receive radiation therapy. The patient remains in clinical remission without evidence of recurrent PTLD, now 3.5 years after completion of all chemotherapy. The liver and small bowel grafts continue to function well without evidence of rejection and low dose IS with sirolimus has been restarted.

## 3. Discussion

PTLD is a serious complication of immunosuppressive therapy after SOT and is a significant cause of morbidity and mortality [2, 3]. The term PTLD includes a spectrum of lesions caused by an uncontrolled proliferation of lymphocytes which is usually associated with Epstein-Barr virus (EBV) infection [4]. Due to the heterogeneity of PTLD and the unique clinical features of each SOT recipient, approaches to both initial treatment and salvage therapy are often individualized. In general, however, in the absence of fulminant disease, treatment typically proceeds in a stepwise fashion, reserving the most intensive therapies for patients with pathologically and clinically aggressive or recurrent disease. Involvement of the CNS is recognized as a particularly poor prognostic feature of PTLD [5, 6]. Reported treatment strategies for CNS PTLD include the use of antiviral agents, immunotherapy, radiation therapy, and chemotherapy, but the outcomes remain dismal [7, 8]. The use of intensive chemotherapy may also pose unique risks to SOT recipients, due to the possibility of chemotherapy-related nephrotoxicity, hepatotoxicity, cardiotoxicity, and/or infectious complications. HD-MTX is an effective chemotherapy agent against lymphoid malignancies and is utilized in the treatment of primary CNS lymphomas and acute lymphoblastic leukemia due to its excellent CNS penetration. Treatment of CNS PTLD with HD-MTX has previously been reported to be efficacious and tolerable in a small number of SOT recipients [9, 10], and we report here our experience using HD-MTX to treat recurrent PTLD of the CNS in a pediatric patient who had received combined liver/small bowel transplantation.

HD-MTX is an effective therapy for CNS PTLD and recurrent PTLD that have failed rituximab and CHOP chemotherapy. With vigilant supportive care to monitor and manage therapy-related complications such as mucositis/colitis, opportunistic infections, and acute rejection, HD-MTX can be safely administered to recipients of liver/small bowel transplantation.
